# Sinomenine Protects against Early Brain Injury by Inhibiting Microglial Inflammatory Response via Nrf2-Dependent Pathway after Subarachnoid Hemorrhage

**DOI:** 10.3390/brainsci13050716

**Published:** 2023-04-25

**Authors:** Chuanjing Fu, Heng Xin, Zhengting Qian, Xiang Li, Juemin Gao, Youwu Fan, Yong Tang, Yan Shi, Ding Li, Heming Wu

**Affiliations:** 1 Department of Neurosurgery, Jiangsu Hospital of Traditional Chinese Medicine, Nanjing 210029, China; 2 Department of Neurosurgery, Nanjing First Hospital, Nanjing Medical University, Nanjing 210006, China; 3 Department of Forensic Medicine, Nanjing Medical University, Nanjing 211166, China

**Keywords:** sinomenine, microglia, subarachnoid hemorrhage, apoptosis

## Abstract

Microglial activation and sustained inflammation plays an important role in the processes of early brain injury (EBI) after subarachnoid hemorrhage (SAH). Sinomenine (SIN) has been demonstrated to have neuroprotective effects in the traumatic brain injury (TBI) model. However, the role of SIN in SAH-induced EBI and its latent mechanisms remain unclear. This study was carried out to explore the role of SIN on SAH-induced EBI and its effects on the microglial inflammatory response following SAH. In this study, a model of SAH in rats was established. Modified neurological severity scores (mNSS), encephaledema, and Nissl staining were employed to determine the effects of SIN. Western blot and immunofluorescence analysis were performed to evaluate nuclear factor erythroid 2-related factor 2 (Nrf2) expression. Nrf2-related downstream proteins, including heme oxygenase-1 (HO-1) and quinine oxidoreductase-1 (NQO-1), were detected with immunohistochemistry analyses and Real-Time Quantitative Polymerase Chain Reaction (RT-qPCR). Microglia activation and associated inflammatory factors, factor-kappa B (NF-κB), interleukin-1β (IL-1β), and interleukin-6 (IL-6), were assessed after SAH. The results showed that SIN administration improved neurobehavior function, and attenuated neural apoptosis and brain edema after SAH. In addition, SIN inhibited microglial action and the subsequent inflammatory response after SAH through the upregulated expression of HO-1 and NQO-1 via activation of the Nrf2 pathway. These results demonstrated that SIN supplementation provided protection against SAH-induced neuronal apoptosis by microglial inflammatory response regulation and possible involvement of the Nrf2 pathway.

## 1. Introduction

Subarachnoid hemorrhage (SAH) is a devastating neurological injury and can have a negative impact on families and society. Early brain injury (EBI) is considered to be the main cause of neurological deficit after SAH. EBI is a complicated complex pathological process that contains oxidative stress, and mitochondrial damage and inflammation, which cause neuronal cell death [1,2]. Inflammation, as an important component in EBI, can be affected by various factors such as ischemia, blood–brain barrier dysfunction, or the activation of microglia. Additionally, the suppression of secondary neuroinflammation has been shown to improve recovery after SAH [3,4]. Therefore, treatment of the microglial inflammatory response has been considered to be the crucial management method following SAH.

Nuclear factor erythroid 2-related factor 2 (Nrf2) is a pleiotropic regulator that plays an important role in modulating the cellular antioxidant reactions, detoxification, and anti-inflammatory responses [5]. Our previous study has shown that Nrf2 exerts neuroprotective effects against TBI by suppressing TBI-induced neuroinflammation [6]. In addition, Nrf2 is considered to be a multiorgan protector and is reported to play an important role after SAH. Under normal circumstances, Nrf2 is coupled with Kelch-like ECH-associated protein 1 (Keap1) and located in the cytoplasm [7]. When exposed to noxious stimulation, the conformation change of Keap1 generates Nrf2 translocation to the nucleus and interacts with the antioxidant responsive element to accelerate phase II enzyme expression, such as heme oxygenase 1 (HO-1), nicotinamide adenine dinucleotide, and phosphate (NADPH)-quinine oxidoreductase1 (NQO-1) [8,9]. However, the mechanism of action of Nrf2 in SAH-induced neuroinflammation remains ambiguous.

Sinomenine (SIN) is the main active ingredient alkaloid extracted from the herb Sinomenium acutum, has a variety of biological functions including anti-inflammatory and anti-neoplastic activities, and cytoprotection, and is mainly used in the treatment of autoimmune and inflammation-associated diseases [10,11]. In addition, SIN has a beneficial effect on different central nervous system diseases, including traumatic brain injury, intracerebral hemorrhage, and Alzheimer’s disease [12,13,14]. Moreover, SIN has the function of regulating the level of antioxidative enzymes as well as preventing various organs’ inflammatory responses after trauma [15]. However, the effects of SIN on SAH-induced EBI are not known, and few studies have involved the effect of SIN on the activation of microglia after SAH. Therefore, the purpose of this study was to investigate the effect of SIN on EBI and the underlying molecular mechanisms after SAH. 

## 2. Materials and Methods

### 2.1. Animals

Experiments protocols were approved by the Committee at Nanjing Medical University and conformed to the Guide for the Care and Use of Laboratory Animals by the National Institutes of Health (NIH). Male Sprague-Dawley rats (280–320 g) were obtained from the Animal Core Facility of Nanjing University of Chinese Medicine. Rats were maintained under comfortable environment at controlled temperature of 26 ± 2 °C with a 12 h light/dark cycle, took standard chow diet and water freely in experimental period. 

### 2.2. Models of SAH 

The SAH endovascular perforation model in rats was performed according to previous article [16]. All of rats were anesthetized via an intraperitoneal injection of 50 mg/kg sodium pentobarbital and maintained anesthesia with 3% isoflurane during the operation. Total of 0.35 mL of nonheparinized fresh autologous arterial blood from the femoral artery was injected (over 20 s) into the prechiasmatic cistern as previous study describes. Sham group received the same procedure with the same dose of saline. The scalp wound was subsequently closed, and rats were returned to quondam cages until recovered from anesthesia. 

### 2.3. Drug Administration

Approximately 30 min after SAH, rats in the SAH + SIN group were intraperitoneally injected with SIN (Sigma-Aldrich, St. Louis, MO, USA), which was dissolved in saline containing 1% dimethyl sulfoxide (DMSO). Rats in the SAH + vehicle group received equal volumes of vehicle (saline containing 1% DMSO) 30 min after SAH after surgery. 

### 2.4. Experimental Design

Design 1: Rats (43 rats were used, 7 rats died) were randomized into six groups (*n* = 6 each): Sham group, SAH, SAH + vehicle, SAH + SIN (20, 50, and 100 mg/kg). All rats were subjected to behavior test assessments before sacrifice for cerebral edema detection. 

Design 2: To determine the effect of SIN after SAH, rats (85 rats were used, 13 rats died) were divided into four groups (*n* = 18 each): Sham group, SAH, SAH + vehicle, SAH + SIN. All animals were sacrificed 24 h after SAH. Assessment methods include Western blot analyses, immunohistochemical staining, Nissl staining, Quantitative Real-Time Polymerase Chain Reaction (RT-qPCR), and immunofluorescence staining.

#### 2.4.1. Brain Water Content

Animals were anesthetized as described above [17]. The brain stem and cerebellum were removed, and the left cerebral hemisphere was separated. The wet weight (ww) of fresh tissue was determined after removing the brain. The hemispheres were parched at 80 °C for 72 h and dry weight (dw) was measured in succession. The percentage of brain water content was calculated by the formula: (ww–dw/ww).

#### 2.4.2. Neurological Evaluation

Neurological deficit was evaluated 24 h after SAH using modified neurological severity score (mNSS) (Table 1). The mNSS score was graded on a scale of 0–18, a total of 18 points indicated severe neurological deficits and normal performance was 0 points, severe injury (13–18 points), mean–moderate injury (7–12 points), and mild injury (1–6 points). The behavioral test was carried out by two independent investigators who were blinded to the experimental groups.

#### 2.4.3. Nissl Staining

Paraffin-embedded sections of brain tissue samples (5 μm thick) were stained with cresyl violet. The sections were hydrated in 1% toluidine blue for 10 min and then dehydrated and fixed with Permount^TM^ mounting medium following by a wash in double-distilled water. The examination was performed by two independent observers who were blinded to the experimental assignments.

#### 2.4.4. Western Blot Analysis

Protein extraction was performed according to the instructions provided by the manufacturer of the Protein Extraction Kit (Beyotime Biotech Inc., Nantong, China). Protein extracts were separated by 10 or 15% sodium dodecyl sulfate-polyacrylamide gel electrophoresis and transferred to polyvinylidene fluoride membranes (Bio-Rad Laboratories, Hercules, CA, USA). The membranes were blocked with 5% nonfat milk for 2 h, and then incubated overnight at 4 °C with Nrf2 (1:1000, Abcam, Cambridge, MA, USA), NF-κB, IL-6, IL-1β (1:400, Cell Signaling Technology, Danvers, MA, USA), histone H3 (1:1000, Abcam, Cambridge, MA, USA), β-actin (1:5000, Bioworld Technology, St. Louis Park, MN, USA), Bax (1:400, Abcam, Cambridge, MA, USA), Bcl-2 (1:200, Santa Cruz, Santa Cruz, CA, USA), Histone H3 (1:1000, Abcam, Cambridge, MA, USA), and cleaved caspase-3 (1:1000, Cell Signaling Technology, Danvers, MA, USA). Subsequently, the membranes were incubated for 2 h with corresponding secondary antibodies conjugated with horseradish peroxidase (1:1000, Bioworld Technology, MN, USA) at room temperature for 2 h. The protein bands were exposed to Tanon-5200 Chemiluminescent Imaging System and strip gray levels were quantified by software (version 4.62; Bio-Rad Laboratories Inc., Berkeley, CA, USA).

#### 2.4.5. Immunohistochemical Staining

Brain tissue samples were fixed in triformol for 24 h and cut at 5 μm thickness, subjected to antigen retrieval in citrate buffer (pH 6.0) for 30 min in a 37 °C chamber, and washed by PBS for three times. The sections were blocked with PBS containing 10% goat serum (Sigma-Aldrich) for 1 h at 37 °C, then incubated with HO-1 (1:200, Abcam, Cambridge, MA, USA), NQO-1 (1:200, 1:200, Abcam, Cambridge, MA, USA) overnight at 4 °C, followed by washing three time in PBS and incubation with horseradish peroxidase (HRP)-conjugated IgG (1:500, Bioworld Technology, MN, USA) for 60 min at room temperature. After three washes for half an hour in PBS, sections were counterstained with hematoxylin, dehydrated, and cleared with xylene before mounting. Control tissue was subjected to the same procedure without the primary antibody step.

#### 2.4.6. Enzyme-Linked Immunosorbent Assay (ELISA)

The peri-contusive cortex was collected and homogenized with lysis buffer and protease inhibitor. The lysate was centrifuged at 12,000× *g* for 20 min at 4 °C. The concentrations of NF-κB, IL-1β, and IL-6 were detected by ELISA kits (Biocalvin Company, Suzhou, China). The OD values of samples were detected by Microplate Reader (MULTISKAN MK3, Thermo, Waltham, MA, USA) and expressed as pg/mg protein.

#### 2.4.7. Immunofluorescence Analysis

Brain tissue samples were embedded in paraffin and cut to 5 μm after fixation in formalin for 24 h. The sections were incubated overnight at 4 °C with primary antibody against Nrf2 (1:50, Santa Cruz Biotechnology, Dallas, TX, USA), Iba-1 (1:50, Santa Cruz Biotechnology), and NeuN (1:100; Boster Biotech, Wuhan, China). After washing three times with PBS, the sections were incubated with secondary antibodies for 2 h at room temperature. Cell nucleus were counterstained with 4′,6-diamidino-2-phenylindole (DAPI) for two min. The sections were covered by coverslip for further study after washing three times. Immunopositive cells were counted using microscope (Leica, Wetzlar, Germany) at ×400 magnification and analyzed by Image Pro Plus 6.0 software (Media Cybernetics, Rockville, MD, USA).

#### 2.4.8. Real-Time Quantitative Polymerase Chain Reaction (RT-qPCR)

Total RNA was extracted from harvested cortex tissues using RNAiso Plus (TaKaRa Bio, Dalian, China). Spectrophotometers and 1% agarose gel electrophoresis assays were used to detect total RNA concentrations. Portions of RNA were reverse transcribed to cDNA with the Prime Script RT reagent kit to avoid RNA degradation. The primer sequences were designed as follows: NQO-1: F: 5′-CAT TCT GAA AGG CTG GTT TGA-3′; R:5′-CTA GCT TTG ATC TGG TTG TCAG-3′; HO-1: F: 5′-ATC GTG CTC GCA TGA ACA CT-3′; R: 5′-CCA ACA CTG CAT TTA CAT GGC-3′; β-actin: F: 5′-AGT GTG ACG TTG ACA TCC GTA-3′; R: 5′-GCC AGA GCA GTA ATC TCC TTCT-3′. The PCR analysis was performed using the Mx3000P System (Strata gene, San Diego, CA, USA). 

## 3. Statistical Analyses

The SPSS 19.0 software(Chicago, IL, USA) was used for statistical analysis. Data are expressed as mean ± SEM and employed ANOVA and Tukey’s post hoc tests. Statistical significance difference was set at *p* < 0.05.

## 4. Results

### 4.1. SIN Ameliorated EBI after SAH in Rats

**Figure 1:**
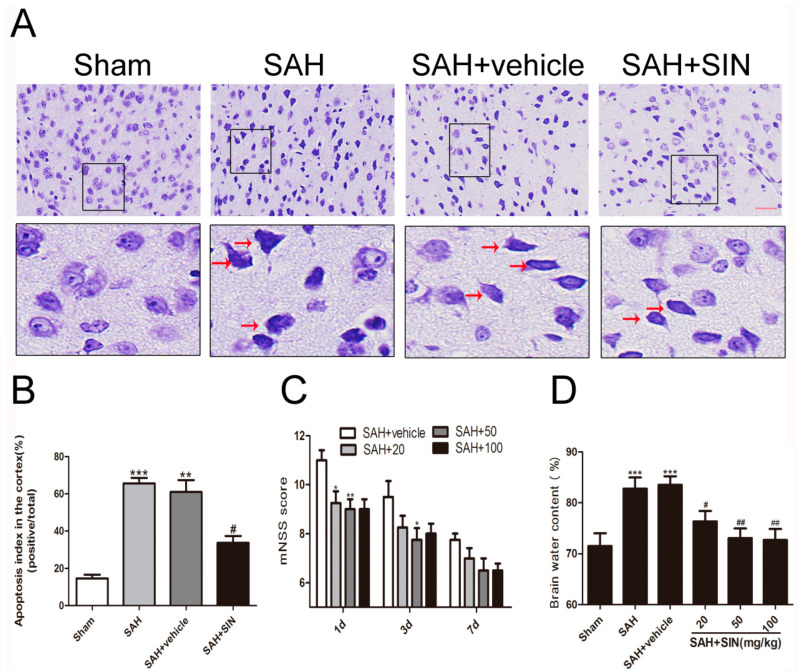
Effects of SIN supplementation on neurological function, brain edema, and neuronal apoptosis after SAH. (**A**,**B**) SIN treatment decreased neuronal apoptosis in rats subjected to SAH. (**C**) SIN treatment improved neurological functions at 1, 3, and 7 days after SAH. (**D**) Brain water content was significantly lower by SIN (20, 50, 100 mg/kg) administration than vehicle-treated group. Values are expressed as mean ± SEM; * *p* < 0.05, ** *p* < 0.01, *** *p* < 0.001 vs. Sham group; ^#^ *p* < 0.05, ^##^ *p* < 0.05 vs. SAH + vehicle. Scale bars = 50 μm (n = 6 per group).

To investigate the effect of SIN on EBI after SAH, brain edema and neurological scores were examined, which revealed that SAH induction aggravated the brain water content and caused neurological impairments compared with the sham group (Figure 1). mNSS was used to assess the neurological function after SAH. As shown in Figure 1C, there were no changes in the sham group over the recorded time span and no difference between SAH and SAH + vehicle groups. Obviously, all groups exhibited improved motor performance with the passage of time after SAH. Within 3 days, the performance of the SAH + vehicle group was significantly better than SAH + vehicle. Higher doses (100 mg/kg) failed to fetch better neurological function scores after SIN treatment. 

As shown in Figure 1D, compared with the Sham group, brain water content increased significantly in the SAH and SAH + vehicle (*p* < 0.05) groups, and there was no obvious difference between the SAH group and SAH + vehicle group. In addition, SIN administration raised the water content of the cerebral cortex after SAH (*p* < 0.05). Compared with the vehicle group, SIN (50, and 100 mg/kg) treatment significantly crippled brain water content. However, a dosage of 100 mg/kg did not represent a better post-SAH protective effect. Thus, 50 mg/kg of SIN was used in the brain edema, neurological score, and the subsequent experiments. 

### 4.2. SIN Decreased Neuronal Degeneration 

As shown in Figure 1A,B, Nissl staining was used to identify neuron apoptosis. Normal neurons had large cell bodies with large round nuclei located in a centrally located soma with a rich cytoplasm. In contrast, damaged cells had shrunken neuronal cell bodies, hyperchromatic nuclei, and dark cytoplasm-containing vesicles, and integrated and clear neurons were found in the brain tissue of the sham group. An abundance of shrinking cells was observed in the SAH and SAH + vehicle groups relative to that in the sham group (*p* < 0.05), while the apoptotic fraction was obviously decreased after SIN treatment (*p* < 0.05). This result indicated that SIN treatment may lead to a reduction in cell apoptosis after SAH.

**Figure 2:**
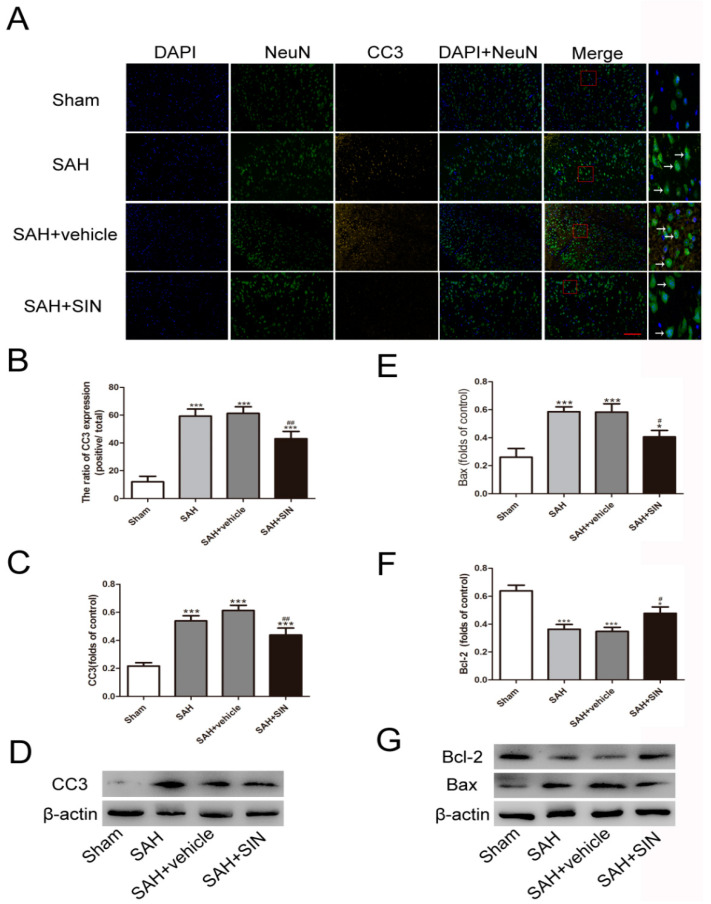
SIN suppresses SAH-induced neuronal apoptosis. (**A**–**D**) Immunofluorescence analysis and Western blot analyses revealed that SAH resulted in the upregulation of CC3, which was decreased after SIN treatment; (**E**–**G**) Western blot analyses revealed Bax levels were decreased, whereas Bcl-2 was increased in TBI + SIN group. Values are expressed as mean ± SEM. * *p* < 0.05, *** *p* < 0.001 vs. Sham group; ^#^ *p* < 0.05, ^##^ *p* < 0.05 vs. SAH + vehicle. Scale bars = 50 μm (n = 6 per group).

As shown in Figure 2, compared with the Sham group, levels of cleaved caspase-3(CC3) and Bax in the SAH and SAH + vehicle groups were significantly elevated (*p* < 0.05), whereas the anti-apoptotic factor Bcl-2 was increased. In addition, Western blot analysis and immunofluorescence analysis showed SIN administration significantly reduced the expression of Bax and CC3 but elevated Bcl-2 in neurons.

### 4.3. SIN Inhibits Microglial Activation and Microglia-Mediated Inflammatory Response after SAH

**Figure 3:**
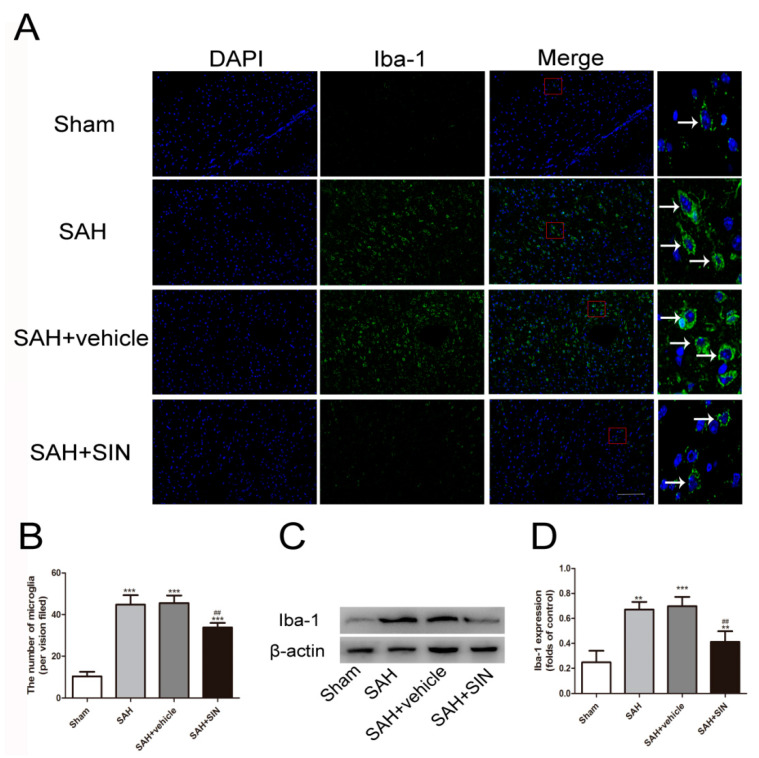
SIN inhibits microglial activation after SAH. (**A**,**B**) Immunofluorescence analysis and (**C**,**D**) Western blot analyses revealed that SAH enhanced the expression of microglial marker, Iba-1, which was significantly decreased by SIN supplementation. Values are expressed as mean ± SEM, ** *p* < 0.01, *** *p* < 0.001 vs. Sham group; ^##^ *p* < 0.05 vs. SAH + vehicle. Scale bars = 50 μm (n = 6 per group).

An effect of SIN treatment on microglial recruitment and the expression of inflammatory factors was detected. As shown in Figure 3, immunofluorescence stain and Western blot analysis revealed that a small number of microglial cells (Iba-1+) were discovered in the Sham group, and were markedly elevated following SAH, which was slashed significantly after SIN administration. These results indicated that SIN could inhibit microglial activation derived from SAH. 

**Figure 4:**
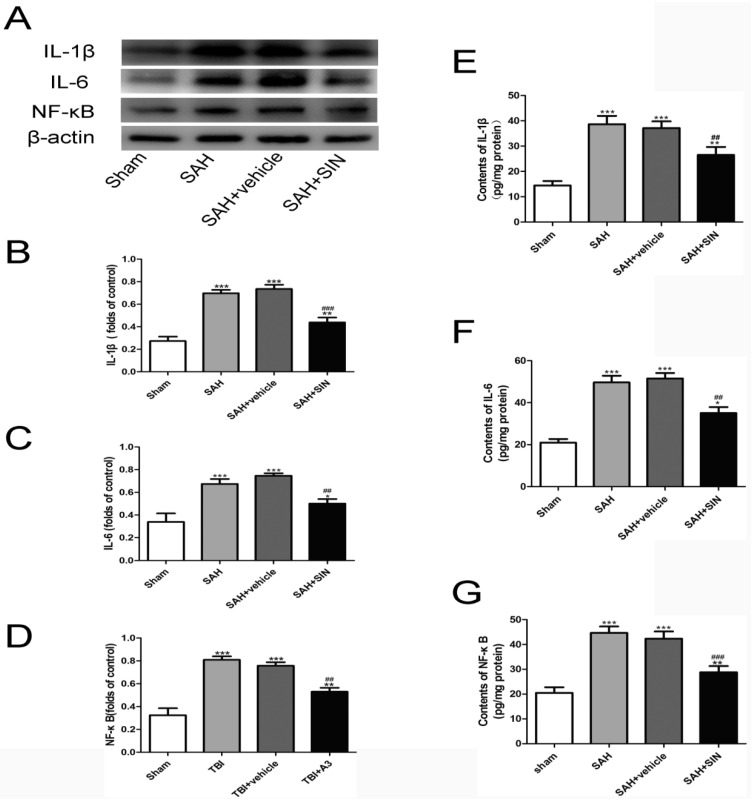
SIN supplementation reduces microglia-mediated inflammatory response after TBI. (**A**–**D**) Expression and quantitative analysis of IL-1β, IL-6, and NF-κB protein in cerebral cortex after SAH. (**E**–**G**) Enzymatic activity of IL-1β, IL-6, and NF-κB in cerebral cortex after SAH. Values are expressed as mean ± SEM. * *p* < 0.05, ** *p* < 0.01 and *** *p* < 0.001 vs. Sham group; ^##^ *p* < 0.01 and ^###^ *p* < 0.001 vs. SAH + vehicle group (n = 6 per group).

To investigate the potential anti-inflammatory role of SIN on microglia, the expressions of pro-inflammatory factors were observed. The alterations in inflammatory factors (NF-κB, IL-1β, and IL-6) were obviously enhanced in the SAH group compared to that of the Sham group, even if their levels were slashed by SIN treatment (Figure 4). 

### 4.4. SIN Promoted Nrf2 Expression and Nuclear Translocation

**Figure 5:**
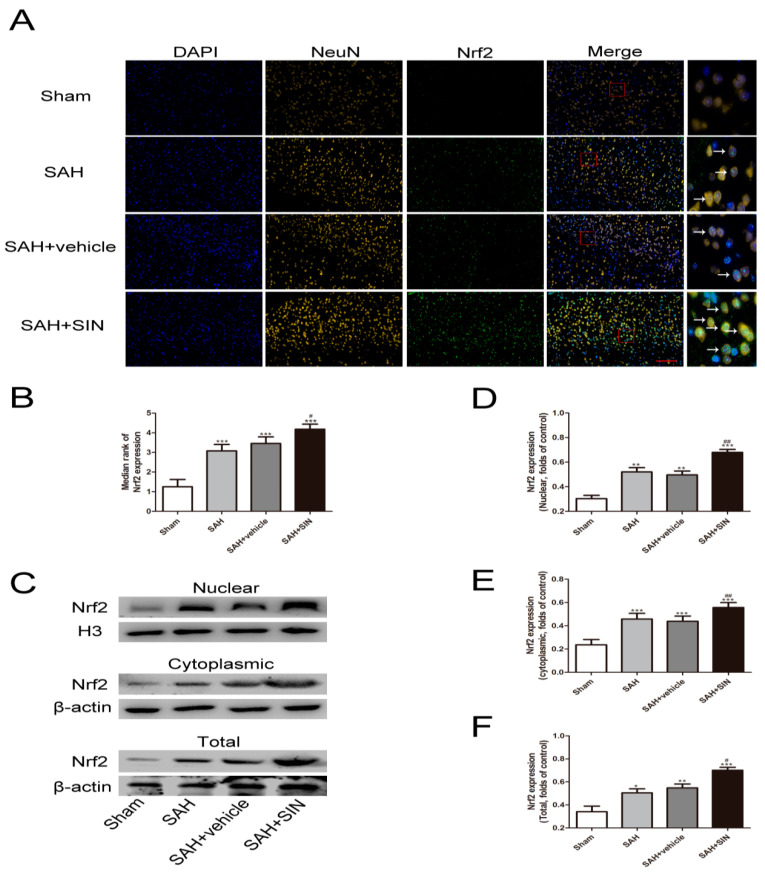
SIN supplementation promotes Nrf2 expression under SAH conditions. (**A**,**B**) Immunofluorescence staining showed SAH enhanced the expression of Nrf2 in neurons, which increased significantly in response to SIN. (**C**–**F**) The nuclear, cytoplasmic, and total SIN expression after SIN treatment with SAH, as measured by Western blot analysis. Values are expressed as mean ± SEM. * *p* < 0.05, ** *p* < 0.01, *** *p* < 0.001 vs. Sham group; ^#^ *p* < 0.05, ^##^ *p* < 0.01 vs. SAH + vehicle group. Scale bars = 50 μm (n = 6 per group).

Nrf2 distribution and expression were investigated by Western blot analysis and immunofluorescence staining. Western blot analysis suggested that increased total and nuclear Nrf2 were observed in the SAH and SAH + vehicle groups compared with the Sham group, and SIN administration showed enhanced expression of total and nuclear Nrf2. Meanwhile, cytoplasmic Nrf2 expression in the SAH + SIN groups were significantly higher than the SAH group (Figure 5). Immunofluorescence staining further confirmed the Western blot analysis results of total Nrf2 and Nrf2 nuclear translocation with SIN treatment in neurons (Figure 5C–F).

### 4.5. SIN Accelerated the Expression of Nrf2 Downstream Factors

**Figure 6:**
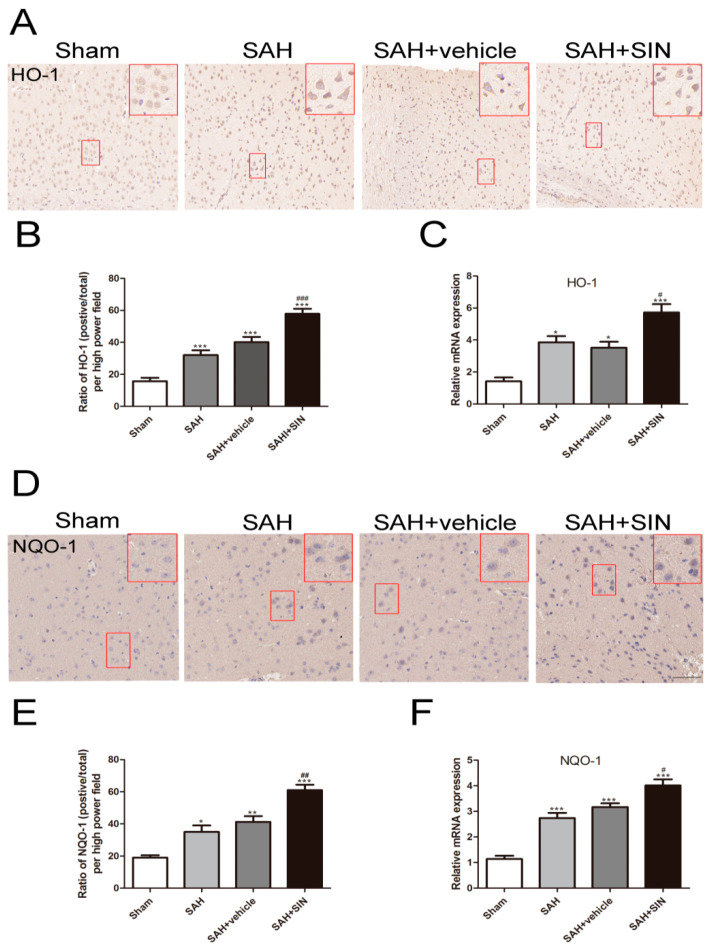
SIN upregulated the expression of HO-1 and NQO-1. (**A**–**C**) Representative immunohistochemical staining images and mRNA levels of HO-1 in cerebral cortex. (**D**–**F**) Representative immunohistochemical staining images and mRNA levels of NQO-1 in cerebral cortex. Values are expressed as mean ± SEM. * *p* < 0.05, ** *p* < 0.01, *** *p* < 0.001 vs. Sham group; ^#^ *p* < 0.05, ^##^ *p* < 0.01, ^###^ *p* < 0.001 vs. SAH + vehicle group. Scale bars = 50 μm (n = 6 per group).

The mRNA and protein levels of NQO-1 and HO-1 were measured as downstream factors of Nrf2 using immunohistochemical staining and RT-qPCR. As shown in Figure 6, NQO-1 and HO-1 protein expression were enhanced significantly after SAH, and SIN further distinctly elevated their expression. Consistent with protein transformation, mRNA results demonstrated that SIN generated greater protein expression of HO-1 and NQO-1 than in the SAH group (Figure 6C,F). These results indicated that SIN induced the expression of HO-1 and NQO-1 at the transcriptional and translational levels.

## 5. Discussion

There is currently no recognized effective treatment for EBI caused by SAH because of its obscure molecular circadian mechanism. The present study demonstrated the neuroprotective effects of SIN of EBI after SAH. Based on the autologous blood injection into the prechiasmatic cistern model in rats, we demonstrated that SIN supplementation reduced brain edema and improved neurological scores. Nissl staining and Western blot analysis showed that SIN supplementation attenuated neuron apoptosis after SAH by inhibiting apoptotic factors, CC3, and Bax expression. It suggested that SIN might have a brain protection effect and improved neurological functions by inhibiting EBI-induced apoptotic factors’ expression.

EBI has complex pathophysiological mechanisms after SAH, which contains oxidative stress, excitotoxicity, and an inflammatory response [18,19]. The inflammatory response is an important step in EBI, which is induced by neuronal necrotic products and hemoglobin degradation products, and stimulates microglia activation after SAH [20]. The activated microglia amplify the inflammatory response, the principle macrophages of the central nervous system, releasing various inflammatory factors and chemokines that further promote neuronal damage [21]. Abundant evidence suggests that microglial activation and the subsequent neuroinflammatory response participates in EBI [22,23]. Our study found that microglia proliferation and subsequent cytokines (NF-κB, IL-1β, and IL-6) were also elevated in the neuronal damaged region after SAH, and were associated with neuron apoptosis. The coincidence of abundant neuronal apoptosis and microglial activation confirmed the important role of the microglia-related neuroinflammation in EBI. 

SIN is an alkaloid derived from Sinomenium acutum with multiple biological activities [24]. Previous studies have demonstrated that SIN can penetrate the blood–brain barrier, which plays a protective effect role in ischemic brain injury and cerebral hemorrhage-induced brain injury by inhibiting microglial activation [25,26]. However, its effects on SAH-induced microglial activation and subsequent inflammation have not been investigated. Our present study demonstrated that SIN supplementation ameliorated the activation of microglia in the injury cortex. Moreover, SIN markedly reduced the expression of NF-κB, IL-1β, and IL-6, which illustrated that SIN could relieve EBI-concomitant inflammation. Consistent with this result, a recent study proved that Nrf2 knockdown in an arthritis mice model reduced the inflammation release effect of SIN, indicating that the anti-inflammatory effect of SIN is dependent on Nrf2 expression [27].

Nrf2 plays a vital role in maintaining cellular homeostasis and metabolism. Numerous evidences have confirmed that it is beneficial to many neurological disorders [28,29,30,31]. Nrf2 binds with Keap1 in the cytoplasm under a physiological state, and noxious stimulation urges it to separate from Keap1 and translocates into the nucleus. A previous study certified that SIN promotes Nrf2 into the nucleus, which triggers the Nrf2-induced anti-inflammatory signaling pathway [32]. Additionally, it is widely accepted that Nrf2 mediates downstream antioxidant enzymes, such as heme oxygenase-1 (HO-1) and NAD(P)H: quinone oxidoreductase-1 (NQO-1) expression after nucleus translocation. Although abundant studies have reported that Nrf2 plays a crucial part in the anti-inflammatory action in a multiple system organ, the effects of SIN on Nrf2 translocation and release have not been elucidated [33]. In this study, we demonstrated that Nrf2 expression and translocation in the cerebral cortex significantly increased after SAH. After that, SIN further enhanced Nrf2 expression and translocation from cytoplasmic to nuclear, while the expression of HO-1 and NQO-1 in neurons increased significantly. These results indicate that SIN ameliorated EBI in an Nrf2-dependent manner. 

There are several potential limitations that should be addressed in our study. First, the role of Nrf2 in modulating the microglia effect by SIN administration was only verified in an in vitro limited sample size. Thus, further cellular and animal experiments are needed to explore the molecular mechanism in the SAH model. Second, we only explored the activated state of Nrf2 after SIN treatment. Thus, the efficient inhibitor might be needed to further explore the mechanism, which should not be ignored in long-term studies. Lastly, the possibility of other properties and signaling pathways that might contribute to the effect of SIN cannot be excluded after SAH. Thus, many more studies are warranted to elaborate on these issues.

## 6. Conclusions

In summary, this study demonstrated that the alkaloid, SIN, inhibited neuronal apoptosis and exerted neuroprotective effects against EBI following SAH. Moreover, SIN increased Nrf2 nuclear translocation and release, thus mitigating the microglial-mediated inflammatory response. These results imply that SIN has a novel therapeutic strategy for EBI after SAH. However, a comprehensive understanding of SIN and its therapeutic implications still requires further investigation.

## Figures and Tables

**Table 1:** Modified neurological severity score.

Motor Tests Points	
Raising rat by the tail	3
1 Flexion of forelimb	
1 Flexion of hindlimb	
1 Head moved > 10° to vertical axis within 30 s	
Placing rat on the floor	3
0 Normal walk	
1 Inability to walk straight	
2 Circling toward the paretic side	
3 Fall down to the paretic side	
Sensory tests	2
1 Placing test (visual and tactile test)	
2 Proprioceptive test (deep sensation, pushing the paw against the table edge to stimulate limb muscles)	
Beam balance tests	6
0 Balances with steady posture	
1 Grasps side of beam	
2 Hugs the beam and one limb fall down from the beam	
3 Hugs the beam and two limbs fall down from the beam, or spins on beam (>60 s)	
4 Attempts to balance on the beam but falls off (>40 s)	
5 Attempts to balance on the beam but falls off (>20 s)	
6 Falls off: No attempt to balance or hang on to the beam (<20 s)	
Reflexes absent and abnormal movements	4
1 Pinna reflex (head shake when touching the auditory meatus)	
1 Corneal reflex (eye blink when lightly touching the cornea with cotton)	
1 Startle reflex (motor response to a brief noise from snapping a clipboard paper	
1 Seizures, myoclonus, myodystony	
Maximum points	18

## Data Availability

The data used to support the findings of this study are available from the corresponding author upon request.
